# Assessment of Knowledge, Attitude and Practice of Dog Owners to Canine Rabies in Wukari Metropolis, Taraba State Nigeria

**DOI:** 10.5539/gjhs.v6n5p226

**Published:** 2014-06-12

**Authors:** Veronica O. Ameh, Asabe A. Dzikwi, Jarlath U. Umoh

**Affiliations:** 1 Department of Veterinary Public Health and Preventive Medicine, Faculty of Veterinary Medicine, Ahmadu Bello University, Zaria, Nigeria

**Keywords:** knowledge, attitude, practice, rabies, vaccination, dog owners

## Abstract

Canine rabies is endemic and occurs throughout the year in all parts of Nigeria. A descriptive cross sectional study was designed to assess knowledge, attitude and practice of dog owners towards rabies, to check for the presence of rabies antigens in brain tissue of dogs slaughtered for human consumption and to assess rabies vaccination coverage of dogs in Wukari. Structured questionnaires were prepared and administered to 200 dog owners by face to face interview. The questionnaire sought information on demographic characteristics of the dog owners, their association with dogs, knowledge, attitude and practice of dog owners towards rabies. Associations between demographic variables and knowledge, attitude or practice scores were assessed using χ^2^ analysis. Also, 188 brain samples from slaughtered dogs were analysed for presence of rabies antigen using direct fluorescent antibody test. Fifteen (7.89%) had rabies antigen. Record files and vaccination certificates of dogs presented to the State Veterinary Hospital Wukari were assessed for anti rabies vaccination coverage. Out of the 200 dog owners, only 26 (13%) knew that rabies virus can be found in nervous tissue, 121 (60.5%) were aware that rabies can be spread through the saliva of a rabid animal, but majority of respondents 172 (86%) did not know the age for first vaccination of dogs against rabies. Dog owners who were civil servants were 4.8 times more likely to have good knowledge (OR=4.84, 95% CI on OR 1.09-21.44) than those of other occupation groups. Positive attitude towards rabies increased with increase in age of dog owners, with respondents within the age group 20-30 years more likely to have negative attitude than those over 40 years. Civil servants were 9.8 times more likely to have good practice than other occupation groups. Rabies antigen was detected in 7.98% of slaughtered dogs. Out of 8370 dogs presented to the hospital between January 2003 and December 2012, only 1128 (13.50%) received anti rabies vaccine. Inadequate knowledge of some aspects of rabies, negative attitude and practice of dog owners towards rabies, the presence of rabies antigen in some dogs slaughtered for human consumption and low vaccination coverage in dogs are indicative of high risk of exposure of dog owners and dog meat processors to rabies. There is therefore a need for educational programmes targeted at dog owners to increase their level of knowledge and reduce the risk of exposure to rabies.

## 1. Introduction

Rabies is a viral disease of all warm blooded animals, which causes acute fatal encephalitis, with almost 100% case fatality rate. This disease occurs in more than 150 countries and territories and about 55 000 people die of rabies every year, mostly in Africa, Asia, and South America (Beard, 2001; WHO, 2006) even though it is a vaccine preventable disease. Forty percent of people who are bitten by suspected rabid animals are children under 15 years of age and dogs are the source of 99% of human rabies deaths (WHO, 2010). Every year, more than 15 million people worldwide receive a post-exposure preventive regimen to avert the disease; this is estimated to prevent 327 000 rabies deaths annually (WHO, 2010).

The disease is endemic in developing countries including Nigeria and other parts of sub-Saharan Africa and Asia (Harry et al., 1984; WHO, 2005) and is often misdiagnosed, under-diagnosed and underreported (Adedeji et al., 2010; Ehizibolo et al., 2011). In Nigeria, the disease was first reported in 1912 in humans, but the first laboratory confirmation was in 1925, by demonstration of Negri bodies in the brain smear of a mad dog (Ekele & Okoh, 1984) and has been declared endemic in Nigeria by several authors (Umoh & Belino, 1979; Fagbami et al., 1981).

Inapparent infection and recovery from clinical disease with resultant persistent or intermittent shedding of rabies virus have affected the overall effort in rabies eradication and control in most parts of the world (Ajayi et al., 2006, Nishizono et al., 2008; WHO, 2005). In Nigeria, rabies infections both in animals and humans are grossly under reported (Fagbami et al., 1981) and reliable data on rabies are scarce in many parts of the world, making it difficult to assess its impact on human and animal health (WHO, 2005). Dog meat is consumed by various ethnic groups in Nigeria. Dog slaughter points are particularly common in Plateau, Cross river, Akwa Ibom, Taraba and Gombe (Simoons, 1994), Kaduna, Kebbi and Ondo (Okonko et al., 2010) and in Abia State (Mshelbwala et al., 2013). Little or no information is available on the epidemiology of rabies in Wukari metropolis even though there is an obvious presence of dogs and dog meat is consumed here. Many dog meat processors and dog owners may not know about the devastating effects of rabies in humans and the potential risk of handling dogs. They may engage in some practices that may therefore expose them to the disease.

Studies on knowledge, attitude and practice (KAP) towards rabies among dog meat processors and dog meat consumers have been carried out in different parts of the country (Opaleye et al., 2006; Isek, 2013; Odeh et al., 2014) and these have shown that KAP is an important factor in the control of rabies in Nigeria.

Vaccination against rabies virus is a highly effective method of preventing the disease in humans and animals (Jakel et al., 2008). Dog rabies control relies on mass vaccination of dogs in order to achieve population immunity levels sufficient to inhibit rabies transmission (Perry & Wandeler, 1993). Mass vaccination has been used successfully in Western Europe and North America (Wandeler, 2000; WHO, 2004), showing that the disease can be controlled and eliminated by vaccination of reservoir animal population. In Nigeria, it has not been possible to successfully control rabies, instead evidences show that the disease is on the increase (Ogunkoya, 1997).

This study was carried out to assess the risk of exposure to rabies by assessing the rabies related knowledge, attitudes and practices of dog owners, checking for evidence of rabies infection in slaughtered dogs and assessing rabies vaccination coverage in dogs in Wukari, Taraba State, Nigeria. 

## 2. Material and Methods

### 2.1 Study Area and Population

This study was carried out in Wukari metropolis of Taraba State, Nigeria. Wukari metropolis is a large town and is the Headquarters of Wukari Local Government Area of Taraba State. The rivers Donga and Benue pass through this area. It shares boundary with Benue State to the south. Geographically, it lies between latitude 7° 53’ 42” North and longitude 9° 47’ 59” East. It is one of the major towns in Taraba State and has an area of 4,308 km² and a population of 241,546 at the 2006 census. It has a State Veterinary Hospital, a General Hospital and other private clinics. The major tribes in the area are Jukun, Kutep, Tiv, Hausa and the Fulani, but Hausa and English are most commonly spoken by all residents.

### 2.2 Study Design and Data Collection

Cross sectional study was carried to assess knowledge, attitude and practice of dog owners and detection of rabies antigen in the brain tissue of dogs slaughtered for human consumption between June to September 2013 in Wukari metropolis, Taraba State, Nigeria. While a descriptive epidemiology was used to assess dog vaccination records in the State Veterinary Hospital Wukari, from January 2003 to December 2012.

### 2.3 Survey Methods

Structured questionnaire was designed then pre- tested employing face to face interview to 10 dog owners within the study area. The questionnaires were analysed and re-evaluated for questions that weren’t clear to the respondents, after wards 200 of the modified questionnaire were distributed to dog owners between June - September 2013. Forty (40) streets, consisting of eight (8) streets in each of the 5 major residential areas (Anguwan dujukun, Filin jirgi, GRA, Pawzu and New Jerusalem) within the study area were surveyed. A total of 200 dog households were surveyed. Starting from the 1^st^ major street of each area, every 2^nd^ street was surveyed using systematic random sampling. In each chosen street, starting from the 1^st^ house, every 2^nd^ house was selected, and an adult member in each household was interviewed using questionnaire. The questionnaire was made up of five (5) sections. The demographic information of respondents was contained in section A. Information on association of respondent with dogs was contained in section B, this had questions on number of dogs owned, period of dog keeping and reasons for keeping dogs. Section C contained information about knowledge of rabies, which included questions on mode of transmission, clinical signs/symptoms and preventive measures. Questions on attitude of respondents towards rabies were provided in section D. Section E contained questions on practice of the respondents towards rabies. The questionnaire was explained to the respondents by the researcher and their responses were recorded. The questionnaires were administered by oral interview only to dog owners who were present and willing to participate at the time of study. The options for the choice questions were “Yes”,” No” and “Don’t know/Undecided”. A marking scheme containing expected correct answers was prepared and used to mark and score the responses. Don’t know/undecided responses were considered as wrong answers. For each correct and incorrect answer, one and zero points were assigned. Houses without dogs or houses that refused to participate in the compound questionnaire survey were skipped for the next houses possessing dog(s). A total of five (5) houses were surveyed in each street chosen.

### 2.4 Detection of Rabies Antigen

One hundred and eighty eight (188) dog heads were purchased from dog meat processors at slaughter points. The brain was removed from each dog head as described by Atanasiu (1975), placed in clean sample bottles and preserved at -20 °C. The samples were then transported on ice packs to the Viral Zoonoses Laboratory of The Department of Veterinary Public Health and Preventive Medicine, Ahmadu Bello University, Zaria, Nigeria. Direct Fluorescent Antibody test (DFA) as described by Dean et al., (1996) was performed on the brain samples collected.

### 2.5 Vaccination Records

Information on age, sex and breed of dogs were collected monthly for the ten year period (2003-2012). Results obtained were presented using tables and charts.

### 2.6 Statistical Analysis

Data obtained was entered into a computer and analyzed using Statistical Package for Social Sciences, SPSS (Version 17, SPSS Inc. Chicago IL USA). Demographic variables were presented using descriptive statistics. The mean knowledge, attitude and practice scores were calculated. Respondents with knowledge, attitude and practice scores equal or greater than the mean scores were considered to have good knowledge, attitude and practice while those who had scores below the mean were categorized as having poor knowledge, attitude and practice. Associations between demographic variables and the categorized scores were assessed using χ^2^ test of association and odds ratio; confidence intervals (95%) were calculated for odds ratios. Values of p<0.05 were considered significant in the χ^2^ analysis. Relationships between non-categorized scores were assessed using multiple regression analysis. Brain samples positive for rabies antigen were expressed as a percentage of the total sample examined. Time series decomposition using ratio to moving average was used to assess seasonal variation in dog vaccination. Time series decomposition using ratio to moving average was used to assess seasonal variation in dog vaccination.

## 3. Results

### 3.1 Demographic Characteristics of the Respondents and Association with Dogs

Out of the 200 respondents that participated in the study, 101 (50.5%) were males. Respondents between the age group 20-30 years were 59 (29.5%) while respondents between were 31-40 years 55 (27.5%). 61 (30.1%) of the respondents were civil servants while 86 (43.0%) were self employed. Based on level of education of respondents, 29 (14.5%) had no formal education while 71 (35.5%) had tertiary education. Majority of respondents 125 (62.5%) were of the Jukun tribe (Table 1).

**Table 1:** Demographic characteristics of respondents in Wukari, Taraba State Nigeria

Characteristics	Total number of respondents N=200	Specific rates (%)
**Gender**		
Male	101	50.5
Female	99	49.5
**Age (years)**		
<19	36	18.0
20-30	59	29.5
31-40	55	27.5
>40	50	25.0
**Marital status**		
Single	67	33.5
Married	133	66.5
**Occupation**		
Unemployed	11	5.5
Civil servant	61	30.5
Self employed	86	43.0
Student	42	21.0
**Educational level**		
No formal education	29	14.5
Primary	42	21.0
Secondary	58	29.0
Tertiary	71	35.5

### 3.2 Association with Dogs

Out of the 200 households visited, a total of 399 dogs were owned by the respondents, with a ratio of approximately 2 dogs per household. Majority of the respondents 190 (95%) kept dogs for protection. Only 23 (11.5%) had specially constructed cages for their dogs and 160 (80%) let their dogs roam freely within the neighborhood. Respondents who reported that they had been previously bitten by a dog were 43 (21.5%) (Table 2).

**Table 2:** Associations of dog owners with dogs in Wukari Taraba State Nigeria

Association item	Total number of respondents N=200	Specific rates (%)
**Number of dogs owned**		
One	68	34.0
Two	90	45.0
Three	29	14.5
>three	13	6.5
**Reason for keeping dogs**		
Protection	190	95.0
Companionship	8	4.0
Hunting	2	1.0
**Period of dog keeping (years)**		
1-5	47	23.5
6-10	40	20.0
11-15	88	44.0
>15	25	12.5
**Dog housing**		
Specially constructed cages	23	11.5
Anywhere on the premises	177	88.5
**Control of dog movement**		
Never allowed to leave the premises	40	20.0
Allowed to roam freely in the neighborhood	160	80.0
**Have you been bitten by a dog**		
Yes	43	21.5
No	157	78.5

### 3.3 Knowledge of Rabies

The mean knowledge score of respondents was 14.30 out of 20 items scored. Majority 171 (85.5%) agreed that rabies does not kill only animals, 26 (13%) knew that the rabies virus can be found in the nervous tissue, 121 (60.5%) agreed that rabies can be spread through the saliva of a rabid animal and 163 (81.5%) agreed that dogs are the possible common source of rabies in Nigeria. One hundred and fifty-nine (71.5%) affirmed that humans can be infected with rabies, but majority of respondents 172 (86%) did not know the age when dogs receive their first dose of anti-rabies vaccine (Table 3). The results show statistical significant association between occupation and knowledge (Table 4), with civil servants 4.8 times more likely to have good knowledge (OR=4.84, 95% CI on OR 1.09-21.44) than other occupation groups (Table 5).

**Table 3:** Assessment of knowledge of the respondents on rabies in Wukari, Taraba State

Knowledge item N=200	Frequency	(%)
**Rabies kills only animals**		
Yes	29	14.5
No	171	85.5
**Rabies virus can be found in the nerves**		
Yes	26	13.0
No	174	87.0
**The virus can be spread through the saliva of a rabid animal**		
Yes	121	60.5
No	79	39.5
**Dogs are common source of rabies in Nigeria**		
Yes	163	81.5
No	37	18.5
**Humans can be infected with rabies**		
Yes	159	79.5
No	41	20.5
**At what age should dogs receive first dose of rabies vaccine?**		
3 months	28	14.0
9 months	172	86.0
**Dog meat processors are at more risk of being infected with rabies virus**		
Yes	54	27.0
No	146	73.0
**A mad dog should not be slaughtered for human consumption**		
Yes	162	81.0
No	38	19.0
**Dog registration and licensing helps in control of rabies**		
Yes	101	50.5
No	99	49.5
**Vaccination of dogs against rabies should be repeated yearly**		
Yes	114	57.0
No	86	43.0

**Table 4:** Association of demographic variables of respondents with categorized knowledge scores of rabies in Wukari, Taraba State

	Categorized scores N=200				
Variables	Poor	Good	*χ^2^*	(df)	p-value
**Age (years)**					
<19	25(69.4)	11(30.6)			
20-30	28(47.5)	31(52.5)	5.384	3	0.146
31-40	27(49.1)	28(50.9)			
>40	24(48.0)	26(52.0)			
**Gender**					
Male	46(45.5)	55(54.5)	3.407	1	0.65
Female	58(58.6)	41(41.4)			
**Marital status**					
Single	40(59.7)	27(40.3)	2.394	1	0.122
Married	64(48.1)	69(51.9)			
**Occupation**					
Unemployed	9(81.8)	2(18.2)			
Civil servant	15(24.6)	46(75.4)	29.304	3	0.000
Self employed	50(58.8)	35(41.2)			
Student	30(69.8)	13(30.2)			
**Educational level**					
No formal education	19(65.5)	10(34.5)			
Primary	25(59.5)	17(40.5)	29.313	3	0.000
Secondary	41(70.7)	17(29.3)			
Tertiary	19(26.8)	52(73.2)			

* *Percentages in parentheses and numbers in front*.

**Table 5:** Multivariable logistic regression analysis of demographic variables and categorized knowledge scores of respondents in Wukari, Taraba State, Nigeria

Variables N=200	Categorized scores N=200				
	Poor	Good	Crude odds ratio (95% CI)	Adjusted odds ratio (95% CI) Poor	Adjusted odds ratio (95% CI) Good
**Age (years)**					
<19	25(69.4)	11(30.6)	2.46(1.00-6.09)	0.32(0.07-1.54)	3.09(0.65-14.76)
20-30	28(47.5)	31(52.5)	0.98(0.46-2.08)	0.42(0.17-1.07)	2.36(0.93-5.97)
31-40	27(49.1)	28(50.9)	1.04(0.49-2.25)	0.84(0.36-1.98)	1.19(0.50-2.82)
>40	24(48.0)	26(52.0)	1	1	1

**Gender**					
Male	46(45.5))	55(54.5))	0.59(0.34-1.03)		
Female	58(58.6)	41(41.4)	1		

**Marital status**					
Single	40(59.7)	27(40.3)	1.60(0.88-2.90)		
Married	64(48.1)	69(51.9)	1		

**Occupation**					
Unemployed	9(81.8)	2(18.2)	1.95(0.37-10.30)	1.40(0.21-9.39)	0.72(0.11-4.81)
Civil servant	15(24.6)	46(75.4)	0.14(0.06-0.34)	0.21(0.05-0.91)	4.84(1.09-21.44)
Self employed	50(58.8)	35(41.2)	0.62(0.28-1.35)	0.27(0.08-0.99)	3.65(1.02-13.12)
Student	30(69.8)	13(30.2)	1	1	1

**Educational level**					
No formal education	19(65.5)	10(34.5)	5.20(2.05-13.16)	4.49(0.94-21.57)	0.22(0.46-1.07)
Primary	25(59.5)	17(40.5)	4.02(1.79-9.05)	4.11(0.90-18.84)	0.24(0.53-1.12)
Secondary	41(70.7)	17(29.3)	6.60(3.05-14.28)	4.52(0.95-21.54)	0.22(0.46-1.05)
Tertiary	19(26.8)	52(73.2)	1	1	1

### 3.4 Attitudes towards Rabies

The mean attitude score was 6.45 out of 9 items, indicating that the respondents had moderately positive attitude towards the disease. Most of the respondents 113 (56.5%) were of the opinion that children should not be allowed to play with dogs, while majority 195 (97.5%) affirmed that they do not play with unknown dogs. Categorized attitude scores showed an association with age, occupation and educational qualification of respondents (χ^2^ = 10.996, df=3, p=0.012, χ^2^ =28.184, df=3, p=0.000 and χ^2^=37.816, df=3, p=0,000 respectively) (Table 6).

**Table 6:** Summary of demographic variables with categorized attitude scores of dog owners in Wukari, Taraba State, Nigeria

Variable	Negative attitude N= 200	Positive attitude	χ^2^	Df	P-value
**Age (years)**					
<19	11(30.5)	25(69.5)			
20-30	30(50.8)	29(49.2)	10.996	3	0.012
31-40	17(30.9)	38(69.1)			
>40	11(22.0)	39(78.0)			
**Gender**					
Male	36(35.6)	65(64.4)	0.118	1	0.731
Female	33(33.3)	66(66.7)			
**Marital status**					
Single	25(37.3)	42(62.7)	0.353	1	0.553
Married	44(38.9)	89(61.1)			
**Occupation**					
Unemployed	6(54.5)	5(45.5)			
Civil servant	6(9.8)	55(90.2)	28.184	3	0.000
Self employed	43(50.6)	42(49.4)			
Student	14(32.6)	29(67.4)			
**Educational level**					
No formal education	18(62.0)	11(38.0)			
Primary	26(61.9)	16(38.1)	37.816	3	0.000
Secondary	14(24.1)	44(75.9)			
Tertiary	11(15.5)	60(84.5)			

* *Percentages in parentheses and numbers in front*.

### 3.5 Practices towards Rabies

Most of the respondents 151 (75.5%) advised that dog handlers should take human anti-rabies vaccine (Table 7). There was statistical significance between the occupation and categorized practice scores of respondent, with civil servants and those who were self employed having higher practice scores (χ^2^=9.983, df=3, p=0.019) than students (Table 8).

**Table 7:** Assessment of practice of respondents towards rabies in Wukari, Taraba State Nigeria

Practice items N=200	Frequency (%)
**It is good to vaccinate your dog**	
Yes	172(86.0)
No	28(14.0)
**Dog handlers should wear protective clothing**	
Yes	167(83.5)
No	33(16.5)
**It is good to wash dog bite wounds with soap**	
Yes	182(91.0)
No	18(9.0)
**Dog handlers should take human anti-rabies vaccine**	
Yes	151(75.5)
No	49(24.5)

* *Percentages in parentheses and numbers in front*.

**Table 8:** Association of demographic variables of dog owners with categorized practice scores in Wukari, Taraba State Nigeria

Variables	Poor Practice N=200	Good Practice	χ^2^	Df	P-value
**Age (years)**					
<19	16(44.4)	20(55.6)			
20-30	23(39.0)	36(61.0)	4.965	3	0.174
31-40	30(54.5)	25(45.5)			
>40	29(50.0)	21(42.0)			
**Gender**					
Male	48(47.5)	53(52.2)	0.178	1	0.673
Female	50(50.5)	49(49.5)			
**Marital status**					
Single	31(46.3)	36(53.7)	0.301	1	0.583
Married	67(50.4)	66(49.6)			
**Occupation**					
Unemployed	7(63.6)	4(36.4)			
Civil servant	20(32.8)	41(67.2)	9.983	3	0.019
Self employed	49(57.6)	36(42.4)			
Student	22(51.2)	21(48.8)			
**Educational level**					
No formal education	19(65.5)	10(34.5)			
Primary	20(47.6)	22(52.4)	7.502	3	0.057
Secondary	32(55.2)	26(44.8)			
Tertiary	27(38.0)	44(62.0)			

* *Percentages in parentheses and numbers in front*.

### 3.6 Detection of Rabies Antigen

Out of the 188 brain samples examined, 15 (7.98%) were positive for rabies antigen. The rate of infection was higher in younger dogs (9, 4.79%) than in older dogs (6, 3.19%). Also, male dogs had higher infection rate 9 (4.79%) than the female dogs 6 (3.19%) (Table 9).

**Table 9:** Sex and age distribution of dogs slaughtered for human consumption in Wukari whose brain tissue samples tested positive for rabies antigen

**Gender**	Age*		**No. Tested (%)**	**No. Positive Sex specific rates (%)**
	**1-24months**	**>24months**		
Male	4/32	5/88	120(63.83)	9(4.79)
Female	5/25	1/43	68(36.17)	6(3.19)
**Total (%)**	**9/57**	**6/131**	**188(100)**	**15(7.98)**

Age* Number positive/Number tested

### 3.7 Vaccination Coverage

**Figure 1:**
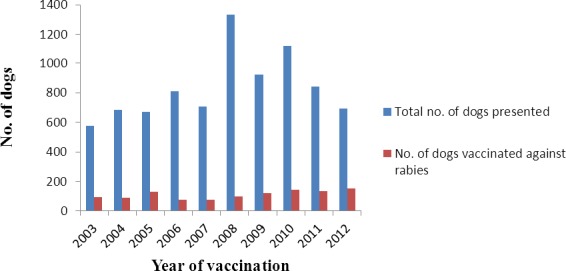
Annual distribution of number of dogs vaccinated against rabies in Wukari from 2003-2012

**Figure 2:**
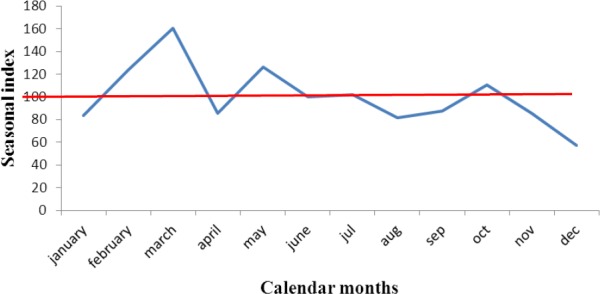
Seasonal variation of vaccinated dogs in Wukari, Taraba State, Nigeria (2003-2012)

A total of 8,370 dog cases were presented to the hospital between January 2003 and December 2012. One thousand one hundred and twenty eight dogs (13.50%) received anti rabies vaccine within this period, 589 (52.2%) males and 539 (47.8%) females. Most of the dogs vaccinated were within 3-12 months of age 664 (58.9%). Indigenous breeds of dog had the highest vaccination coverage 921 (81.6) compared with exotic breeds and crosses (Table 10). There was an increase in vaccination rates of dogs in the years 2005, 2009, 2010, 2011 and 2012 (Figure 1) as compared with other years. Seasonal index of vaccination of dogs within Wukari peaked in the month of March (Figure 2).

**Table 10:** Sex, breed and age distribution of dogs vaccinated against rabies in State Veterinary Hospital Wukari, Taraba State

Gender	No. of dogs vaccinated	Percentage (%)
Male	589	52.2
Female	539	47.8
**Breed**		
Indigenous	921	81.6
Exotic	76	6.7
Cross	131	11.6
**Age**		
3-12months	664	58.9
12-36	385	34.1
>36	79	7.0
**Total**	**1128**	**100**

## 4. Discussion

Dog owners in this study showed an acceptable level of knowledge on mode of transmission of rabies, clinical signs of the disease and effects of licensing and registration of dog, but had poor knowledge of age of first vaccination of dogs, with a mean knowledge score of 14.30. Most of the respondents did not agree that dog meat processors were at risk of being exposed to the disease. There was statistical significant association between occupation, educational level and level of knowledge of respondents, with civil servants and those with secondary and tertiary education having higher knowledge scores. This may be because they have higher educational background than those in other occupational categories in this study. The findings agree with study done by Isek (2013). Age of respondents also had an effect on the level of knowledge. Dog owners within the age group 20-30 were more likely to have poor knowledge of the disease than older respondents.

Respondents’ attitude of not playing with unknown sick dogs, not allowing stray dogs to roam freely in their compounds, seeking medical assistance if bitten by a dog and not allowing children play with dogs are good signs of the people’s involvement in the control of rabies. Positive attitude of respondents towards the control of rabies increased with age.

Practices of good vaccination of dogs, advising dog handlers to wear protective clothing and take human anti-rabies vaccine and washing of dog bite wounds with soap and water are also indicators that the community is involved in the control of the disease. Poor practices of using traditional medicine and non-washing of dog bite wounds goes against the WHO recommendation of instituting medical treatment on victims of dog bite. These negative practices may be as a result of inadequate awareness of the possible dangers of rabies. Individuals involved in the handling and processing of dog meat are constantly being exposed to rabies virus through bites from dogs, cuts or wounds that are not well protected from infective tissue or saliva of these dogs, thereby constituting a great risk of rabies exposure. Presence of antigens to rabies in brain of dogs slaughtered for human consumption is very significant in the epidemiology of the disease. This means that dog meat processors and handlers are at risk of being exposed to rabies either from bites from dogs before slaughter or by coming in contact with infective tissue or saliva. This agrees with previous studies carried out in different locations in Nigeria (Garba et al., 2008; Akombo, 2009; Aliyu et al., 2010; Isek, 2013). Result of this study shows that more male dogs and younger dogs (1-24 months) were positive for rabies than the females and older dogs. This may be because more male dogs were presented for slaughter than female dogs at the time of this study. This disagrees with the study carried out by Baba (2006), Aliyu et al. (2010) and Isek (2013) which showed that more adult dogs tested positive for rabies antigen compared to younger dogs.

The vaccination profile indicates that indigenous breed of dogs had higher vaccination coverage (81.6%) than exotic breeds and crosses. This can be attributed to the high population of indigenous breeds in the area compared to exotic breeds and crosses which are more expensive to acquire and maintain. This is in agreement with the study carried out by Isek (Isek, 2013) Dogs within the age group 3-12 months had the highest vaccination coverage (58.9%), compared to older dogs. this may be due to frequent advice to dog owners to vaccinate their dogs from 3 months of age and above. Dogs less than 6 months are very important in transmission of rabies in humans and are more likely to be accessible for parenteral vaccination than older dogs (Mitmoonpitak et al., 1998). The increase in number of dogs vaccinated in recent years (2009-2012) (Figure 1) may be as a result of increased awareness of dog owners on the need to vaccinate their dogs and the possible dangers of rabies to humans and animals. The peak in dog vaccination in March (Figure 2), this might be connected to findings in studies carried out by Owai (2009), Umoh and Belino (1979) and Tomori (1980) who reported that most outbreaks of rabies occur in the dry season which coincides with the breeding season of dogs in most parts of Nigeria. Presence of these outbreaks might stimulate dog owners to vaccinate their dogs against rabies during this period.

The presence of antigen to rabies virus (7.98%) in brain tissues of dogs slaughtered for human consumption is a clear indication of the presence of rabies virus and plays a significant role in the epidemiology of the disease in the area. Factors such as lack of awareness on the availability of human anti-rabies vaccines, low vaccination coverage in dogs, non-wearing of protective clothing by dog meat processors, dog bites and exposure of cuts or wounds to saliva and brain tissue during dog meat processing all put dog meat processors and dog owners at high risk of being exposed to rabies virus.

## References

[ref1] Adedeji A. O., Eyarefe O. D., Okonko I. O., Ojezele M. O., Amusan T. A., Abubakar M. J. (2010). Why is there still rabies in Nigeria? A review of the current and future trends in the epidemiology, prevention, treatment, control and possible eradication of rabies. British Journal of Dairy Sciences.

[ref2] Ajayi B. B., Rabo J. S., Baba S. S. (2006). Rabies in Apparently Healthy Dogs Histological and Immunological Studies. The Nigerian Postgraduate Medical Journal.

[ref3] Akombo P. M. (2009). Dog Ecology and Epidemiological Studies of Canine Rabies in Benue State.

[ref4] Aliyu T. B., De N., Yenda E. N., Lynn M. (2010). Prevalence of Rabies virus antigens in Aparently Healthy Dogs in Yola, Nigeria. The Research.

[ref5] Atanasiu P., Baer G. M., Annual inoculation and the Negri body (1975). the natural history of rabies (pp. 373-400).

[ref6] Baba S. S. (2006). Detection of rabies virus RNA and Antigen in tissues from naturally infected Nigerian Dogs;In-situ hybridization and immunohistochemical studies. Reveu d’Elevage et de Medicine Veterinaire des pays Tropicaux.

[ref7] Beard M. (2001). Woman Dies of Rabies after Nigerian Dog Bite. Independent Newspaer, The London, June 1 2001.

[ref8] Dean D. J., Abelseth M. K., Atanasiu P., Meslin F. X., Kaplan M. M., Koprowski, The fluorescent antibody test (1996). Laboratory Techniques in Rabies.

[ref9] Ehizibolo D. O., Ehizibolo P. O., Ehizibolo E. E., Sugun M. Y., Idachaba S. E. (2011). The control of neglected zoonotic diseases in Nigeria through animal intervention: an overview. African Journal of Biomedical Research.

[ref10] Ekele A., Okoh J. (1984). Rabies in a Vaccinated Dog in Plateau State *Nigeria Bulletin*. Animal Production.

[ref11] Fagbami A. H., Anosa V. O., Ezebiuro E. O. (1981). Hospital Records of Human Rabies and Antirabies Prophylaxis in Nigeria 1969-1978. Transactions of the Royal Society of Tropical Medicine and Hygiene.

[ref12] Garba A., Oboegbulem S. I., Elsa A. U., Junaidu A. W., Magaji A. A., Umoh J. U., … Y., Masdooq A. A. (2008). A Comparative Rabies Laboratory Diagnosis: Peculiar features of samples from apparently healthy dogs in Nigeria. Sokoto Journal of Veterinary Sciences.

[ref13] Harry T.O., Adeiga A., Anyiwo C. E., Nasid A. (1984). Anti-rabies treatment of dog bite Victims in Lagos, Nigeria: Trial of Suckling Mouse Brain and Fetal Bovine Kidney cell rabies Vaccinee. Vaccine.

[ref14] Isek T. I. (2013). Epidemiological Studies of Canine Rabies in Ogoja, Cross river State, Nigeria.

[ref15] Jakel V., Konig M., Cussler K., Hanschmann K., Thiel H. J. (2008). Factors Influencing the Antibody Response to Vaccination against Rabies. Development Biology (Basel).

[ref16] Mitmoonpitak C., Tepsumethanon V., Wilde H. (1998). Rabies in Thailand. Epidemiology and infection.

[ref17] Mshelbwala P. P., Ogunkoya A. B., Maikai B. V. (2013). Detection of rabies antigen in the saliva and brains of apparently healthy dogs slaughtered for human consumption and its public health implications in Abia State, Nigeria. ISRN Veterinary Science.

[ref18] Nishizono A., Khawplod P., Ahmed K., Goto K., Shiota S., Mifune K., Morimoto K. (2008). A simple and rapid immunochromatographic test kit for rabies diagnosis. Microbiology and immunology.

[ref19] Odeh L. E., Umoh J. U., Dzikwi A. A. (2014). Assessment of Risk of Possible Exposure to Rabies among Processors and Consumers of Dog Meat in Zaria and Kafanchan, Kaduna State, Nigeria. Global Journal of Health Science.

[ref20] Ogunkoya A. B. (1997). Rabies: Basic concepts, problems and prospects of its control in Nigeria. Oreofe Nigeria Limited.

[ref21] Okonko I. O., Adedeji O. B., Babalola E. T., Fajobi E. A., Fowotade A., Adewale O. G. (2010). Why Is There Still Rabies in the World?-An Emerging Microbial and Global Health Threat.

[ref22] Opaleye O. O., Adesiji Y. O., Olowe O. A., Fagbami A. H. (2006). Rabies and antirabies immunization in South Western Nigeria: knowledge, attitude and practice. Tropical doctor.

[ref23] Owai P. U. (2009). A Study of Rabies in dogs in Calabar, Cross River State, Nigeria. Journal of Applied Science.

[ref24] Perry B. D., Wandeler A. I. (1993). The delivery of oral rabies vaccines to dogs: an African perspective. The Onderstepoort Journal of Veterinary Research.

[ref25] Simoons F. J. (1994). Eat not this flesh: food avoidances from prehistory to the present. Univ of Wisconsin Press.

[ref26] Tomori O. (1980). Wild life rabies in Nigeria: experimental infection and transmission studies with the shrew (Crocidura sp.. Annals of tropical medicine and parasitology.

[ref27] Umoh J. U., Belino E. D. (1979). Rabies in Nigeria. A historical review. International journal of zoonoses.

[ref28] Wandeler A. I. (2000). Oral immunization against rabies: afterthoughts and foresight. Schweizer Archiv fur Tierheilkunde.

[ref29] WHO W. (2005). Technical Report Series 931. WHO EXPERT CONSULTATION ON RABIES, First Report.

[ref30] WHO W., WHO Technical Report Series (2005). WHO Expert Consultation on Rabies, 2004.

[ref31] WHO W. (2006). World Survey on Rabies No. 42 for the year 2006.

[ref32] WHO W. (2010). Rabies Fact Sheet 99.

